# Cell-Free DNA Provides a Good Representation of the Tumor Genome Despite Its Biased Fragmentation Patterns

**DOI:** 10.1371/journal.pone.0169231

**Published:** 2017-01-03

**Authors:** Xiangyuan Ma, Liangjun Zhu, Xue Wu, Hua Bao, Xiaonan Wang, Zhili Chang, Yang W. Shao, Zhenxin Wang

**Affiliations:** 1 Geneseeq Technology Inc., Suite 300, MaRS Centre, South Tower, Toronto, Ontario, Canada; 2 Jiangsu Cancer Hospital, 42 Baiziting, Xuanwu, Nanjing, Jiangsu, China; 3 Nanjing Shihe Jiyin Biotechnology Inc., 17th Floor, Building B, Sino-Danish Life Science Park, 3 Xinjinhu Road, Nanjing, Jiangsu, China; 4 Department of Medical Oncology, The First Affiliated Hospital of Soochow University, Cang Lang Qu, Suzhou, Jiangsu, China; University of Navarra, SPAIN

## Abstract

Cell-free DNA (cfDNA) is short, extracellular, fragmented double-stranded DNA found in plasma. Plasma of patients with solid tumor has been found to show significantly increased quantities of cfDNA. Although currently poorly understood, the mechanism of cfDNA generation is speculated to be a product of genomic DNA fragmentation during cellular apoptosis and necrosis. Sequencing of cfDNA with tumor origin has identified tumor biomarkers, elucidating molecular pathology and assisting in accurate diagnosis. In this study, we performed whole-genome sequencing ofcfDNA samples with matching tumor and whole blood samples from five patients diagnosed with stage IV gastric or lung cancer. We analyzed the coverage spectrum of the human genome in our cfDNA samples. cfDNA exhibited no large regions with significant under-coverage, although we observed unbalanced coverage depth in cfDNA at transcription start sites and exon boundaries as a consequence of biased fragmentation due to ordered nucleosome positioning. We also analyzed the copy number variant status based on the whole-genome sequencing results and found high similarity between copy number profile constructed from tumor samples and cfDNA samples. Overall, we conclude that cfDNA comprises a good representation of the tumor genome in late stage gastric and lung cancer.

## Introduction

The presence of double-stranded cell-free DNA (cfDNA) in healthy human plasma has been noted since 1948[1]. Under conditions such as pregnancy, autoimmune disorders, myocardial infarction, and cancer, the concentration of cfDNA in plasma is significantly increased[2–5].Although highly variable between patients and cancer types, the plasma concentration of cfDNA has also been found to strongly correlates with the stage of cancer[5,6]. The exact mechanism by which cfDNA is released into circulation is still unclear; however, many have speculated that it is generated by apoptotic or necrotic cells[7,8]. Supporting this hypothesis, the size distribution of cfDNA peaks at ~170 bp, which is reminiscent of single-nucleosomal DNA fragments generated during apoptosis[9–11].Recently, Snyder and colleagues identified bias in cfDNA fragmentation pattern using whole-genome sequencing (WGS)[12]. This bias is a result of nucleosome positioning and transcription factor binding which protect DNA from nuclease digestion during apoptosis, leaving footprints in cfDNA that inform its tissue-of-origin[12].

Analysis of molecular biomarkers obtained through tissue biopsy or surgical resection can elucidate the molecular pathogenesis of cancer, providing foundation for accurate clinical diagnosis. Sometimes, a patient’s physical condition does not permit the collection of a tumor sample through invasive methods; in contrast, collection of cfDNA involves minimal invasiveness. In addition, a single biopsy is generally insufficient to represent the spatial and temporal heterogeneity displayed within most tumors[13]. On the contrary, cfDNA has been reported to show fast clearance from circulation and carries markers of its source tissue[12,14,15]. Thus, due to the clinical advantages, cfDNA has attracted much attention for the purpose of monitoring disease progression and treatment efficacy in the past decade. Recent advancement in DNA detection technology has expanded analysis of cfDNA from basic properties, such as concentration and fragment size, to complex features such as nucleotide sequences in various cancer types[14,16–22]. Compared to the polymerase chain reaction (PCR)-based detection method, which examines defined mutations at a specific genomic locus, next-generation sequencing (NGS)-based techniques permit profiling of cfDNA at a broader range[23–26]. WGS has been deployed at low coverage depth (0.1X-10X) to successfully identify copy number variation (CNV) and structural variation (SV) in cfDNA[27–29].Single nucleotide variations (SNV) and small insertion/deletion (indel) mutations can be further identified by targeted sequencing or whole-exome sequencing at relatively higher coverage depth[26,28,30].

Beyond the footprints left behind by transcription factors binding and nucleosome positioning[12], we wondered whether cfDNA displays further bias in coverage of genomic regions. To investigate whether cfDNA fully represents the whole human genome, we performed WGS on the cfDNA samples with matching tumor DNA samples and whole blood DNA samples collected from five cancer patients. We examined the uniformity of cfDNA coverage over the whole genome and the whole exome in a detailed manner. We validated that WGS of cfDNA with low average coverage depth (~10X) is sufficient to detect CNVs identified in matching tumor samples. Moreover, we identified specific characteristics in cfDNA fragmentation pattern near genomic features such as transcription start sites (TSS) and exonic boundaries, where nucleosome positioning is highly phased. In conclusion, our results demonstrate that cfDNA is a good representation of the whole genome and a comparable resource to primary tumor DNA for clinical applications.

## Results and Discussion

### General features of cfDNA

Patients recruited in this study were diagnosed with either late stage gastric cancer or late stage lung cancer with various level of metastases (Table 1). From the five plasma samples, an average ranging from 19.6 ng to 172.8 ng of cfDNA was extracted from 1ml of plasma (S1 Table), comparable to previously reported value [5]. Of note, two processing methods were used for tumor sampleT03 (body fluid effusion): namely, conventional genomic DNA extraction from the cell portion (“T03N”) and cfDNA extraction method from the liquid portion(“T03S”) (Table 1).A significantly higher amount of cfDNA was extracted from T03S compared to plasma samples, yielding 6516.7ng cfDNA per ml of body fluid. Whole-genome sequencing libraries were constructed following protocols according to sample types (see METHODS). An average of 405.7 million sequencing reads was obtained per sample, approximately corresponding to 10-fold depth of coverage of the human reference genome(Table 1).

**Table 1:** Patient and sample information.

Patient ID	Gender	Cancer Type	Stage	Metastasis	Sample ID	Sample Type	Total Reads (Million)	Alignment rate	Read length	Mean Coverage depth (X)
P1	Female	Gastric Cancer	IV	-	B01	Whole Blood	398.5	99.63%	PE-75^‡^	9.40
					T01	Fresh Tissue Biopsy	313.7	99.79%	PE-75^‡^	7.62
					C01	Plasma	320.7	99.67%	PE-75^‡^	7.42
P2	Male	Lung Adenosquamous Carcinoma	IV	Lymph Node Metastasis	B02	Whole Blood	324.2	99.52%	PE-75^‡^	7.48
					T02	FFPE^†^	486.1	99.59%	PE-75^‡^	10.92
					C02	Plasma	395.7	99.55%	PE-75^‡^	9.23
P3	Female	Gastric Cancer	IV	-	B03	Whole Blood	465.0	99.66%	PE-75^‡^	10.86
					T03S	Body Fluid*	433.6	99.71%	PE-75^‡^	9.38
					T03N	Body Fluid**	444.3	99.67%	PE-75^‡^	10.40
					C03	Plasma	437.3	99.68%	PE-75^‡^	10.36
P4	Female	Lung Adenocarcinoma	IV	-	B04	Whole Blood	410.9	99.71%	PE-75^‡^	9.70
					T04	Fresh Tissue Biopsy	461.1	99.73%	PE-75^‡^	10.37
					C04	Plasma	386.8	99.70%	PE-75^‡^	8.24
P5	Male	Lung Adenocarcinoma	IV	Liver and Brain Metastasis	B05	Whole Blood	502.3	99.60%	PE-75^‡^	11.71
					T05	FFPE^†^	289.7	97.30%	PE-75^‡^	6.05
					C05	Plasma	421.1	99.61%	PE-75^‡^	9.65

* DNA was extracted from the clear fluid of the sample after centrifugation following plasma DNA extraction protocol.

** DNA was extracted from the cell pellet of the sample after centrifugation

† FFPE: Formalin-fixed, paraffin-embedded

‡ PE-75: Paired-end, 75 base pairs

**Fig 1:**
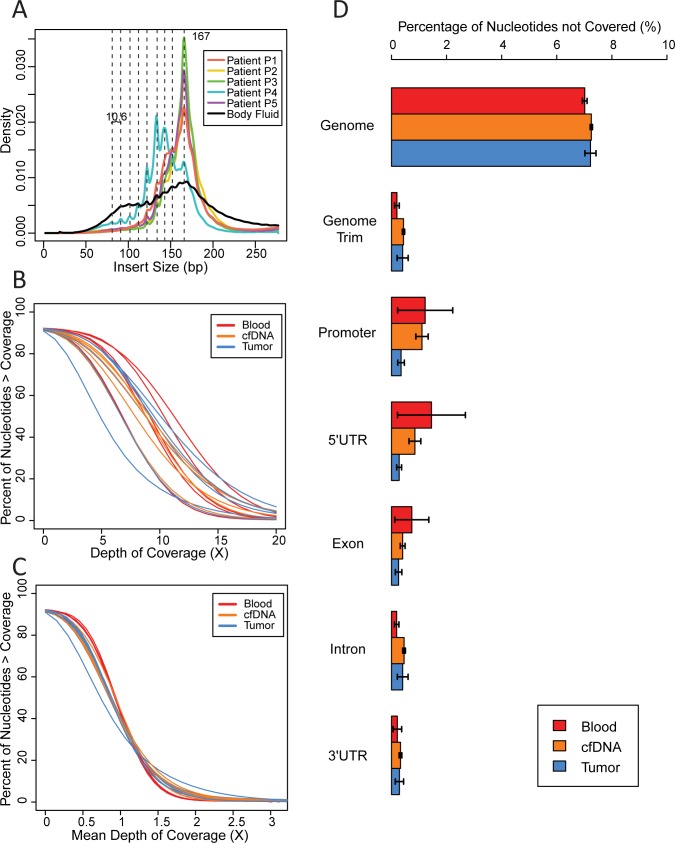
cfDNA fragment size distribution and coverage uniformity. (A) Fragment size distribution of cfDNA extracted from plasma or the liquid portion of body fluid effusion samples. Vertical dashed lines mark local maxima. (B) Cumulative plot of percentage of nucleotides covered by a specific depth. (C) Cumulative plot of percentage of nucleotides covered by a specific depth after normalization to global mean depth of coverage of that sample. (D) Percentage of nucleotides showing no coverage genome wide and in promoter regions, 5’ UTR, exons, introns, and 3’ UTR. Annotation of the hg19 reference genome was obtained from the UCSC table browser. Genomic Trim represented hg19 reference genome after all “N” nucleotides was removed. Each bar represents the mean of 5 biological replicates for blood and tumor samples and 6 biological replicates for cfDNA samples. Error bars represents standard error. No changes between groups were statistically significant as tested by one-way ANOVA.

We first examined the size distribution of cfDNA by analyzing insert length from the sequencing data(Fig 1 and S1 Fig). Consistent with previous findings[12,31], the majority of cfDNA samples showed a predominant peak at 167bp, with multiple local maxima between the size of 80bp and 167bp (Fig 1A). We calculated the mean inter-peak distances between the local maxima to be 10.6bp, which is comparable with previously reported values[12].Blood samples and most tumor samples showed normal distribution in insert size due to mechanical shearing of genomic DNA, with peak sizes dependent on sample preparation procedure (S1A and S1B Fig).

cfDNA extracted from the body fluid of Patient P3 showed a distinct size distribution compared to that isolated from the same patient's plasma (Fig 1A and S1C Fig), with a noticeable fraction of inserts at around 330bp, resembling dinucleosomal DNA. Patient P4’s cfDNA sample exhibited severe fragmentation with dominant peaks at 133bp and 144bp (Fig 1A), while her fresh tumor sample showed a bimodal distribution pattern peaking at around 165bp and 330bp, with additional local maxima less than 330bp (S1B Fig). Interestingly, Patient P4’s cfDNA size distribution was reminiscent of a recent report claiming that cfDNA fragments of tumor origin are shorter in length[32]. Closer investigation uncovered contradictory results in the literature regarding correlation of cfDNA fragment integrity to cancer progression. On one hand, increased amounts of long cfDNA fragments have been detected in patients with advanced tumor, while on the other hand fragment integrity was found to be lower in mice with greater tumor burden in a colorectal cancer mouse xenograft model[33,34].We postulate that the shorter size and bimodal distribution pattern of Patient P4’s cfDNA is a consequence of high tumor burden. Additional cases and experiments will be required to fully establish this hypothesized correlation between shorter cfDNA size, bimodal tumor DNA fragment size distribution, tumor burden, and degree of necrosis within the tumor.

### Coverage of the whole-genome in cfDNA

Next, we plotted the percentage of the genome covered within our sample against raw (Fig 1B) or normalized (Fig 1C) cumulative depth of coverage. All samples achieved around 90% genome coverage by at least 1X depth and displayed a sigmoid trend. Blood samples showed a better performance with slower entry into and steeper slope within the linear range of the curves. Tumor samples and cfDNA samples showed similar performances. The tumor sample with the fastest drop in percent genome coverage was T05, which was sequenced at a lower mean coverage of 6.05X. We then calculated the percentage of nucleotides that failed to be covered by at least 1X depth within several types of genomic features, including promoter region (see METHODS for definition), 5’ untranslated region (UTR), exonic region, intronic region, and 3’ UTR (Fig 1D). One-way ANOVA failed to detect any differences between sample types. We also examined the size and the position of base pairs displaying no coverage in any of the five samples (S2 Fig). Although certain genomic positions showed large regions without coverage, which appeared as vertical clusters of dots in (S2C Fig), this pattern is not a unique characteristic of cfDNA samples, and is also present in tumor and whole blood.

### Hierarchical clustering distinguishes samples by patient

**Fig 2:**
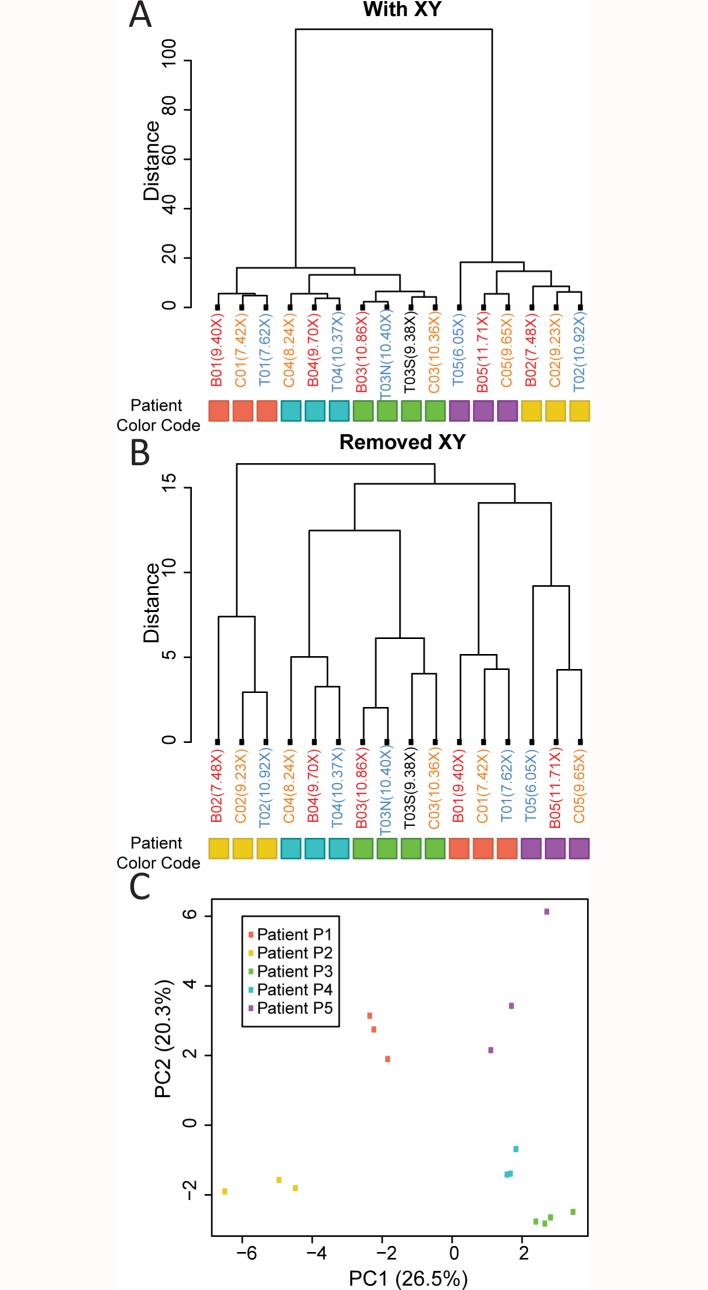
Hierarchical clustering and PCA of the fraction of nucleotides covered in each 10kbp window. (A) Unsupervised hierarchical clustering using the fraction of nucleotides covered in each consecutive, non-overlapping 10kbp window. Coloration of each leaf was based on sample types: red for blood, blue for tumor, orange for plasma cfDNA, and black for body fluid effusion cfDNA samples. Number in brackets in each leaf indicated sample mean coverage depth. (B) Unsupervised hierarchical clustering using the fraction of nucleotides covered in each consecutive, non-overlapping 10kbp window, with data from chromosome X and Y removed. Coloration of each leaf is based on sample types: red for blood, blue for tumor, orange for plasma cfDNA, and black for body fluid effusion cfDNA samples. Numbers in brackets in each leaf indicate sample mean coverage depth. (C) PCA on the fraction of nucleotides covered in each consecutive, non-overlapping 10kbp window with chromosome X and Y data removed. Features were not scaled to equal variance.

If cfDNA displays biased coverage that is unique to the sample type, analyzing the sequencing data using hierarchical clustering should cluster all cfDNA samples together. To test this hypothesis, we divided the human reference genome into consecutive, non-overlapping 10k bp windows, and calculated the percentage of nucleotides covered in each 10k bp window for all samples. Hierarchical clustering was performed as shown in Fig 2A and 2B. Interestingly, regardless of sample type and mean depth of coverage, 16 samples formed five major clusters, each representing an individual patient. The five clusters are separated into two major branches of the dendrogram (Fig 2A), which can be readily explained by the gender of the patients. However, even after removing sex chromosomes, the clustering that separates patients persists. Principle component analysis (PCA) using the same data confirmed the observation. On a plot of principle component (PC) 2 versus PC1, data points are clustered together based on patients rather than sample types or sequencing depth (Fig 2C).

Correct grouping of cfDNA samples to their matching tumor samples was not unexpected, since CNV influences the percentage of nucleotides with coverage within a 10kbp window, and CNV was ubiquitously found throughout the genome while being unique in pattern to each patient. It is surprising however that blood samples, which serve as the germ-line control for each patient, were not grouped together. Most samples from the same patient are collected at the same time. However, library preparation of different sample types followed distinct protocols handled by different operators. Some libraries were even sequenced multiple times and the results were pooled to reach desired sequencing depth. Batch-to-batch variations should be minimized during the experimental procedure. We therefore believe correct grouping of blood samples to their matching cfDNA and tumor samples is a consequence of germ-line insertion/deletion mutations unique to each patient.

### Patient copy number variation profile

**Fig 3:**
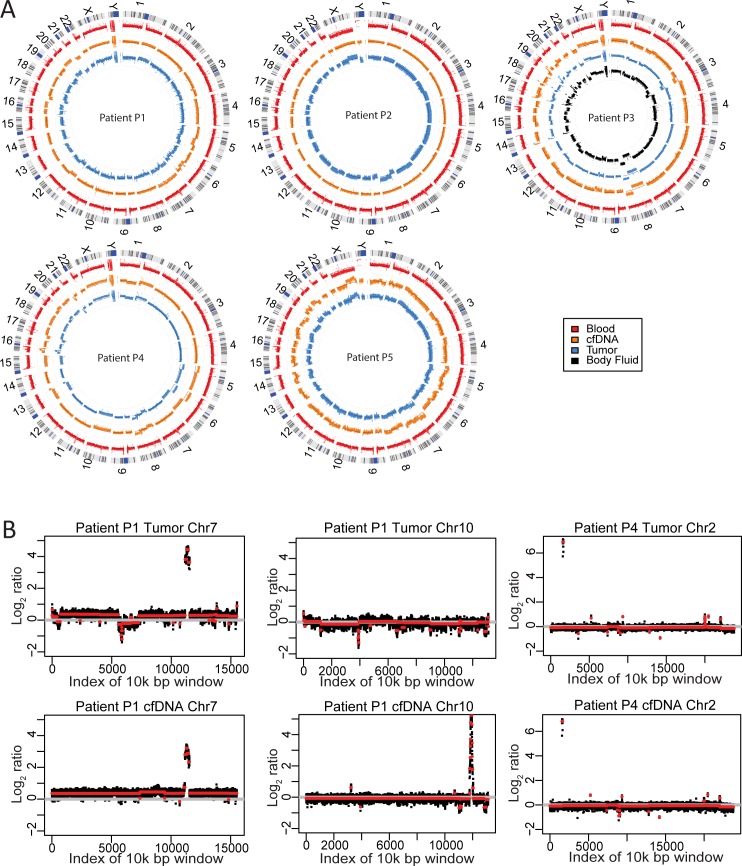
Construction of the CNV profile of each patient. (A) Circos plots showing the CNV profile of each patient. The outermost ring plots the ideogram of autosome 1–22 and chromosome X and Y of the hg19 reference genome. Blue segments on the ideograms correspond to unmappable regions of hg19. Each ring plots the log2 ratio in the consecutive, non-overlapping 10kbp window of a sample. Log2 ratios for blood samples were calculated against publicly available NA18535 WGS results. Log2 ratios for cfDNA, tumor, and body fluid samples were calculated against their respective blood sample. (B) Segmented CNV profiles of selected chromosomes. The x-axis represented the 10kbp window index. The y-axis represented log2 ratio. The grey horizontal line marks the theoretical 2 copy state. The red line represented the calculated copy number status of that segment.

Multiple studies have successfully demonstrated that WGS of plasma cfDNA samples at low mean depth (0.1X to 10X) is sufficient to detect CNV[27–29]. We tallied the read count mapped to each consecutive non-overlapping 10k bp window in the reference human genome for each sample. After normalization to GC content in each window by LOESS (S3 Fig) and to mean depth of coverage, we were able to generate the CNV profile for each patient by plotting the log_2_ ratio between cfDNA or tumor data and blood data. The log2 ratios for blood samples were calculated using whole-genome sequencing data of NA18535 (Chinese Han female) from the 1000 Genome Project[35]. Increased and decreased log2 ratio in a CNV profile represents copy number gain and loss of a chromosomal region, respectively. Neglecting the sex chromosomes and regions near centromeres, blood sample CNV profiles demonstrated small variance from the baseline centered at 0, which corresponds to 2 copies (Fig 3A). In contrast, CNV profiles of cfDNA and tumor displayed parts of or whole chromosomal regions that deviated from the baseline, excepted for cfDNA of patient P2. The concentration of cfDNA extracted from Patient P2 was 19.6 ng/ml plasma, a concentration much lower than that of the other cfDNA samples we extracted (S1 Table), and barely higher than reported value from healthy individuals[11]. It is plausible that cfDNA of tumor origin constitutes only a limited percentage of Patient P2’s total cfDNA, resulting in its baseline-like CNV profile.

We also calculated pair-wise Spearman’s correlation coefficient between samples using their GC-content-normalized read counts in consecutive non-overlapping 10kb windows. The calculated correlation coefficients were plotted in a heat map shown in S4 Fig. The correlation coefficients suggest overall similarity between the genomic regions that display amplification or deletion, with 1 and 0 corresponding to exact correlation and complete irrelevance, respectively. Most cfDNA samples displayed greater than 0.8 Spearman’s correlation coefficient to their respective tumor sample, while generally exhibited less than 0.6 Spearman’s correlation coefficient to their respective blood sample or samples from other patients. Patient P2 was an exception, largely due to the fact that his cfDNA CNV profile showed little abnormality compared to blood.

Segmentation of the CNV profile allowed detection of gene-level amplifications such as *c-MET* amplification (chromosome 7q31.2) in patient P1 and *MYCN* amplification (chromosome 2p24.3) in patient P4 (Fig 3B). Interestingly, we noticed a roughly 20X amplification of *FGFR2* gene (chromosome 10q26.13) in patient P1’s cfDNA sample but not in her fresh tumor biopsy sample. A deeper investigation into patient P1’s treatment history showed that the cfDNA sample was collected roughly 5 months after her tumor sample (S1 Table). Between the time points when her tumor and cfDNA samples were taken, the patient undertook Crizotinib treatment, a small-molecule inhibitor for ALK and MET[36], to target her c-*MET* amplification. The patient displayed temporary shrinkage in tumor size and relief of symptoms, but the tumor quickly developed resistance and progressed before the cfDNA sample was taken.

The absence of *FGFR2* amplification in patient P1's tumor sample can be explained in two ways. First, it is possible that cancer cells harboring the*FGFR2* amplification represent only a sub-population of the whole tumor. This sub-population could either be of low abundance, and therefore below the detection limit, or technically easy to miss with the fine needle biopsy. Second, it is possible that *FGFR2* amplification is a *de novo* mutation developed after Crizotinib treatment. *FGFR2* amplification is frequently found in gastric cancer patients, but is often mutually exclusive to *c-MET* amplification [37,38]. Both genes belong to the receptor tyrosine kinase family and participate in similar signaling pathways[38]. Supporting this hypothesis, increased FGFR2 expression has been reported after cell lines harboring *c-MET* amplification gained resistance to small chemical inhibitors[39,40]. In both possible scenarios, cfDNA has the potential to perform better than tumor biopsy at representing the tumor spatial and temporal heterogeneity.

### Analysis of cfDNA fragment boundary captured nucleosome footprints

**Fig 4:**
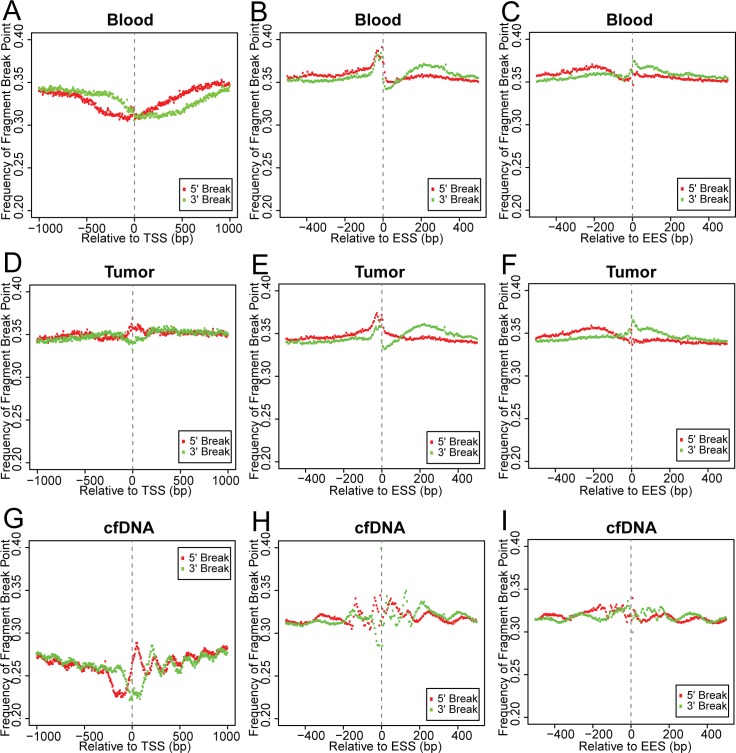
Fragment boundaries inherit nucleosome footprints. (A-C) Plot of 5’ and 3’ fragment boundary frequencies in consecutive non-overlapping 5bp windows within ±1000bp of the TSS (A) and ±500bp of the ESS (B) and the EES (C) in blood samples. (D-F) Plot of 5’ and 3’ fragment boundary frequencies in consecutive non-overlapping 5bp windows within ±1000bp of the TSS (D) and ±500bp of the ESS (E) and the EES (F) in tumor samples.(G-I) Plot of 5’ and 3’ fragment boundary frequencies in consecutive non-overlapping 5bp windows within ±1000bp of the TSS (G) and ±500bp of the ESS (H) and the EES (I) in cfDNA samples.

It has been hypothesized that cfDNA is generated during the process of apoptosis or necrosis, when genomic DNA is digested by a nuclease. Nucleosome-bound genomic DNA is protected from nuclease digestion and thus producing DNA fragments of mono-nucleosomal length. Previous study on nucleosomal positioning in the human genome showed phased placement near TSS[41]. In order to determine whether nucleosome positioning does indeed lead to biased fragmentation patterns in cfDNA samples, we counted the number of reads originating from and terminating at each 5bp window within the upstream and downstream 1000bp region of each TSS(corresponding to the 5’ and 3’ boundary of each DNA fragment, respectively).Frequency of fragmentation was then calculated by dividing the counts by total number of regions examined (Fig 4). In general, the fragment break points showed no observable correlation with respect to the positioning of nucleosomes, although blood samples displayed a reduction in 5' and 3' boundaries at the TSS and tumor samples displayed a slight gain in 5' boundaries at the TSS (Fig 4A and 4D). In contrast, the distribution of break points near the TSS in cfDNA samples showed strong phases (Fig 4G).Regions showing high or low frequency of fragmentation correspond to the nucleotides between or occupied by nucleosomes, respectively. The distance between the 5’ break point and its immediate downstream 3’ break point is roughly 180bp, which is longer than the mode of cfDNA fragment size (167 bp) and coincides with the length of mono-nucleosomal DNA. We are able to identify up to 4 and 5 nucleosomes upstream and downstream of the TSS, respectively, as well as a region devoid of nucleosome binding immediately upstream of the TSS.

Positioning of nucleosomes at exon/intron boundaries is also highly phased[42]. We performed similar analysis for the upstream and downstream 500bp of exon start sites (ESSs) and exon end sites (EESs). Blood and tumor samples displayed increased 5' and 3' fragmentation immediately before and reduced 3’ fragmentation immediately after the ESS (Fig 4B and 4E). On the other hand, cfDNA samples displayed a phased fragmentation pattern at the ESS, with 4 sharp peaks within the +/- 200bp from the ESS, and 2 minor peaks further upstream and downstream (Fig 4H). Interestingly, the inter-peak distance within +/- 200bp around the ESS is smaller than the length of single-nucleosomal DNA, suggesting additional factors affect the cfDNA fragmentation pattern around the ESS. Additionally, up to 4 nucleosome binding sites can be identified from the phased fragmentation pattern near the EES in cfDNA but not in blood and tumor samples (Fig 4C, 4F and 4I).The nucleosome binding sites uncovered from this analysis of break point peaks also matches reported nucleosome positioning at these genomic features[41–43]. This observation provided further evidence that apoptosis and necrosis constitutes the mechanism of cfDNA generation.

### Biased fragmentation interfered with coverage uniformity

**Fig 5:**
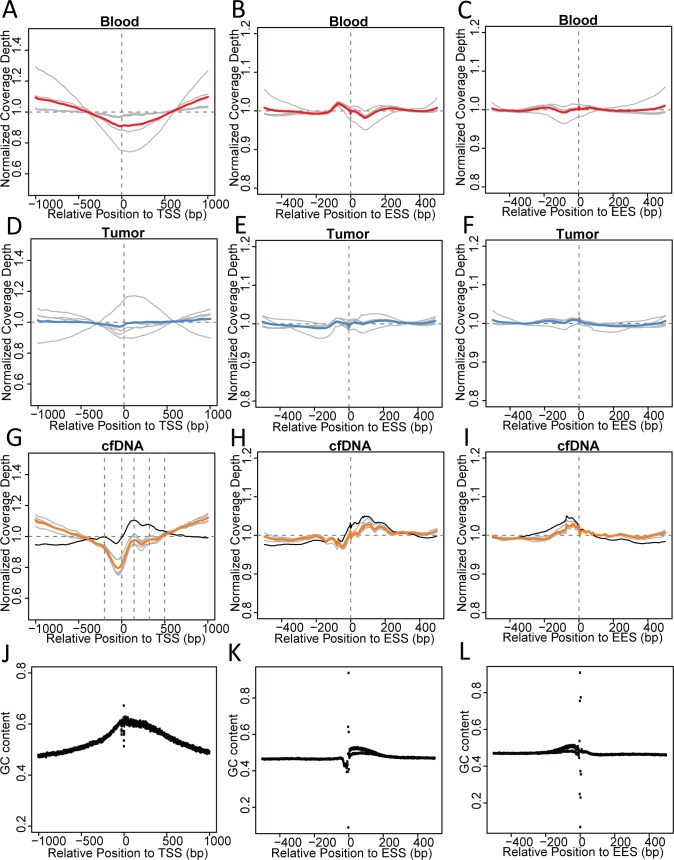
Nucleosome footprints interfered with cfDNA WGS sequencing uniformity. (A-C) Coverage depth at each nucleotide normalized to regional mean depth within ±1000bp of the TSS (A) and ±500bp of the ESS (B) and EES (C) in blood samples. Each grey line represents a sample. Red line represented the mean value of all samples. (D-F) Coverage depth at each nucleotide normalized to regional mean depth within ±1000bp of the TSS (D) and ±500bp of the ESS (E) and EES (F) in cfDNA samples. Each grey line represents a plasma cfDNA sample. Black lines represents the body fluid cfDNA sample. Orange line represents the mean value of all plasma cfDNA samples. Vertical dashed lines mark local coverage depth maxima. (G-I) Coverage depth at each nucleotide normalized to regional mean depth within ±1000bp of the TSS (G) and ±500bp of the ESS (H) and EES (I) in tumor samples. Each grey line represents a sample. Blue lines represent the mean value of all samples. (J-L) Per base GC content within ±1000bp of the TSS (J) and ±500bp of the ESS (K) and the EES (L).

Since biased fragmentation patterns could affect the uniformity of genome coverage in cfDNA samples, we examined the depth of coverage near the TSS, ESS, and EES. Fig 5 plotted the coverage depth at each nucleotide near the TSS, ESS, and EES normalized to the mean depth of the region. Coverage depth displayed slightly lower than mean at TSS while gradually increasing as the nucleotide is further away in blood and tumor samples (Fig 5A and 5D). This pattern highly resembles what was observed in WGS data of randomly selected samples from the 1000 Genome Project[35] (S5 Fig). cfDNA samples also displayed lower than mean coverage depth at the TSS, as well as consistent under coverage at a short region upstream, corresponding to the region devoid of histone binding (Fig 5G). The position of the first three nucleosomes downstream of the TSS and one upstream of the TSS are also in phase with local increases in coverage depth in cfDNA samples. Near the ESS and EES, blood and tumor samples displayed uniform coverage, except for the gain and loss of depth immediately upstream and downstream of the ESS, respectively (Fig 5B, 5C, 5E and 5F). cfDNA samples showed increased depth downstream of the ESS and upstream of the EES, in phase with the fragmentation pattern (Fig 5H and 5I). These positions of gained coverage depth are in line with reported nucleosome occupancy after the ESS and before the EES[42,43], but the span of each peak within the region of depth gain was only half of mononucleosomal DNA length, a possible consequence of differentially phased nucleosomes.

Analysis of the GC content near these genomic features revealed that the imbalanced coverage in blood and tumor samples was in negative correlation with GC content (Fig 5J, 5K and 5L), consistent with reported trend [44].However, the coverage depth bias in cfDNA samples was insufficiently explained by GC content alone. Although the bias in fragmentation and coverage depth was consistently observed in cfDNA samples, we found no evidence suggesting that the biased fragmentation impaired mutation detection at specific genomic loci or in specific genes. As demonstrated in S2 Fig, we didn’t identify any large genomic regions lacking coverage unique to cfDNA. Additionally, the bias in fragmentation only contributes to an approximately 20% decrease in sequencing depth at the nucleosome depleted region immediately upstream of TSS, where the strongest bias was observed.

## Conclusions

In summary, we compared sequencing uniformity in WGS data of matched cfDNA, tumor, and blood sample from five late stage cancer patients. We provided experimental evidence of biased fragmentation at genomic regions near TSS, ESS, and EES. In cfDNA samples, nucleotides displaying frequent fragmentations exhibited decreased coverage depth, to roughly 80% of the regional mean depth. Despite that, biased fragmentation did not contribute to similarities among cfDNA samples identifiable by hierarchical clustering or PCA. At the gene level, biased fragmentation and coverage depth did not impair the detection of CNV mutations in large genomic regions in cfDNA samples. We would like to call researcher’s attention to the biased coverage when utilizing cfDNA to analyze genomic regions that harbor highly phased nucleosomes. However, cfDNA is still a powerful tool when surveying biomarkers in patients with malignancy, and serves as a good surrogate to FFPE sample or fresh tissue biopsy.

## Material and Methods

### Patient enrollment and sample collection

This study was approved by the ethics board of the First Affiliated Hospital of Soochow University and Jiangsu Cancer Hospital of China. Written consent was also obtained from each patient to allow the use of their samples for scientific research. The five patients enrolled in this study came from the Chinese Han ethnic group and were recruited from different hospitals across China during December 2014 and August 2015 (see S1 Table for detailed dates).Each patient’s samples were collected within the same month except for Patient P1, whose peripheral blood and tumor samples were collected December 2014 while the cfDNA sample was collected May 2015. 5–10 ml of peripheral blood was collected from each patient and placed in EDTA-coated tubes (BD Biosciences). Plasma separation was performed within 2 hours of blood collection by centrifuging 5ml whole blood at 1800rcf at 4°C for 10min. All fresh samples were shipped to the central testing laboratory (Nanjing Shihe Jiyin Biotechnology Inc., Nanjing, China) within 48 hours from sample collection. Formalin fixed paraffin embedded (FFPE) blocks/sections or fresh tumor tissues/biopsies were obtained from the hospitals, after examination by pathologists for diagnosis and tumor purity.

### DNA extraction and quantification

cfDNA was extracted with NucleoSpin Plasma XS kit (Macherey Nagel) using a customized protocol optimized based on the manufacturer’s instructions. Fresh tissue DNA and whole blood DNA were extracted using DNeasy Blood & Tissue kit (QIAGEN) following the manufacturer’s protocols. FFPE samples were de-paraffinized with xylene and DNA was extracted using QIAamp DNA FFPE Tissue Kit (QIAGEN) according to the manufacturer’s protocols. For the body fluid effusion sample, the cell portion was extracted following the protocol for fresh tumor and the liquid portion was extracted following the protocol for cfDNA extraction. Purified DNA was qualified by Nanodrop2000 (Thermo Fisher Scientific) and quantified by Qubit 2.0 using the dsDNA HS Assay Kit (Life Technologies) according to the manufacturer’s recommendations. DNA concentration measurements were tabulated in S1 Table.

### Library preparation

Sequencing libraries were prepared with KAPA Hyper Prep kit (KAPA Biosystems) with customized protocol optimized based on the manufacturer’s instructions. In brief, 1 μg of genomic DNA sheared into 350 bp fragments using the Covaris M220 instrument (Covaris), or 2ng-100ng of cfDNA, were processed by end-repairing, A-tailing and ligation with indexed adapters compatible with the Illumina sequencing platform (Illumina), followed by size selection using AMPure XP beads (Agencourt), PCR amplification with Illumina p5 (5'-AAT GAT ACG GCG ACC ACC GA 3') and p7 (5'-CAA GCA GAA GAC GGC ATA CGA GAT 3') primers, and purification by AMPure XP beads.

### Sequencing

Quantification of libraries was performed by quantitative polymerase chain reaction (qPCR) using the KAPA Library Quantification kit (KAPA Biosystems). Library fragment size was determined by the Agilent 2100 Bioanalyzer (Agilent Technologies). All sequencing was performed on the Illumina HiSeq4000 NGS platform (Illumina) using paired-end 75bp sequencing chemistry.

### Sequence data processing

Trimmomatic [45] was used for FASTQ file quality control (QC). Leading/trailing low quality (quality reading below 15) or N bases were removed. Reads from each sample were mapped to reference sequence hg19 (Human Genome version 19) using Burrows-Wheeler Aligner (BWA MEM) [46] with default parameters. Only chromosome 1 to 22, X, Y, and mitochondria were kept in the reference genome. PCR duplicates were removed using Picard Tools (available at: http://picard.sourceforge.net) with default parameters.

### Calculating per-base coverage

The genomic coordinates of 5’ UTR, 3’ UTR, exons, introns, and TSS were obtained from the UCSC table browser [47]. Promoter region is defined as the upstream and downstream 2000 bp of the TSS. Coverage at each nucleotide was calculated using SAMTools [48] and customized bioinformatic scripts. Sex chromosomes were excluded in this analysis.

### Hierarchical clustering and PCA

The humanhg19 reference genome was separated into consecutive, none-overlapping 10k bp windows. Percentages of nucleotides with at least 1X coverage depth were calculated for each window. Distance for hierarchical clustering was calculated using Euclidean method and clustered using Ward.D2 method available in R. PCA was performed without scaling each feature to equal variance, since percentage of covered nucleotides for a 10k bp window could only take values between 0 and 1.

### CNV calculation

The humanhg19 reference genome was separated into consecutive, none-overlapping 10k bp windows. The read count mapped to each window were determined by BEDTools [49]. Normalization of read counts by GC-content was performed using LOESS method with R programming language as previously described [44,50]. After normalization by GC-content and global mean depth of coverage, log2 ratio for each 10k bp window were calculated by dividing NA18535 WGS sequencing data for blood samples, or by dividing their respective blood sample for cfDNA and tumor samples. Segmentation of the log2 ratio data was performed using a circular binary segmentation algorithm.

### Mapping fragmentation points near TSS, ESS, and EES

The 5’ and 3’ boundary of each read near TSS, ESS, and EES were determined by customized bioinformatic scripts similar to published methods[41]. Sex chromosomes were excluded in this analysis. Briefly, paired reads mapped to the regions 1000bp upstream and downstream of the TSS or 500 bp of the ESS and EES were extracted. After adjusting for gene strandness, the number of read pairs originating from and terminating at each consecutive, none-overlapping 5bp window was tallied. Read pair count in each 5bp window was normalized to the total number of TSS, ESS, or EES analyzed.

## Supporting Information

S1 Table Additional patient and sample information. (PDF)

S1 Fig WGS insert size distribution. (A) Fragment size distribution of DNA extracted from blood samples. (B) Fragment size distribution of DNA extracted from fresh tumor, FFPE, or the cell portion of body fluid effusion samples. (C) Fragment size distribution of DNA extracted from plasma or the liquid fraction of body fluid effusion samples. This graph displays size distribution up to 400 bp. (D) Fragment size distribution of all cfDNA samples pooled together. Vertical dashed lines mark local maxima.
(PDF)

S2 Fig Position and size of fragment not covered. Plots showing the size and position of nucleotide fragments not covered in any of the (A) blood samples, (B) tumor samples, or (C) cfDNA samples. The x-axis displays genomic position. The y-axis displays the length of the no-coverage fragments in log_10_ scale. Vertical gray dashed lines marked the boundary between chromosomes. Sex chromosomes were excluded from this graph.
(PDF)

S3 Fig Read count normalization to GC content by locally weighted scatterplot smooth (LOESS). GC content and read counts in each consecutive non-overlapping 10kbp window of the human reference genome hg19 were calculated. After removing regions with extreme read count values (> 99.9% percentile), LOESS was fitted to the scatterplot. Each graph demonstrates the read count spanning 25% to 70% GC content of a sample before or after the normalization. Red line represents the fitted LOESS model.
(PDF)

S4 Fig Similarity between the CNV profiles. The human reference genome hg19 was divided into consecutive non-overlapping 10kbp windows. Sequencing read count mapped to each window were tallied. After removing sex chromosomes and regions with extreme read count values (>99.9% percentile) and normalizing read count to GC content, pair-wise calculation of Spearman correlation coefficient between the CNV profiles was performed. The results were plotted in this heat map. The same color was used to label all samples collected from the same patient.
(PDF)

S5 Fig Normalized coverage depth near the TSS, ESS, and EES in the 1000 Genome Project. Coverage depth at each nucleotide normalized to regional mean depth within ±1000bp of the TSS and ±500bp of the ESS and EES in randomly selected samples from the 1000 Genome Project. Each grey line represents a sample. Red lines represent the mean value of all samples.
(PDF)
