# Supplementary material for: Cell-Free DNA Provides a Good Representation of the Tumor Genome Despite Its Biased Fragmentation Patterns
Source: PLoS One. 2017 Jan 3;12(1):e0169231. doi: 10.1371/journal.pone.0169231 (PMC5207727; doi:10.1371/journal.pone.0169231)
Supplement: S1 Table — (PDF) [file pone.0169231.s001.pdf]

S1 Table. Additional patient and sample information

Patient ID	Gender	Cancer Diagnosis	Treatment History	Sample ID	Collection Date (YY/MM/DD)	Total DNA Extracted (ng)	Input Plasma or Body Fluid Volume (μl)	cfDNA Concentration (ng/ml plasma)
P1	F	Stomach Cancer (Stage IV)	Oxaliplatin, Tegafur+Gimeracil+Oteracil, Docetaxel, Irinotecan, Pemetrexed, Etoposide, Cyclophosphamide	B01	14/12/04	6048		
				T01	14/12/04	4960		
				C01	15/04/08	139.04	1200	115.9
P2	M	Lung Adenosquamous Carcinoma (Stage IV), with Lymph Node Metastasis	Gemcitabine, Cisplatin, Erlotinib, Etoposide	B02	15/04/20	12040		
				T02	15/04/05	7360		
				C02	15/04/20	32.3	1650	19.6
P3	F	Stomach Cancer (Stage IV)	None	B03	15/07/01	9120		
				T03S	15/07/01	15640	2400	6516.7
				T03N	15/07/01	6560		
				C03	15/07/01	691.2	4000	172.8
P4	F	Lung Adenocarcinoma (Stage IV)	Gefitinib	B04	15/07/09	8720		
				T04	15/07/09	8560		
				C04	15/07/09	134	2400	55.8
P5	M	Lung Adenocarcinoma (Stage IV), with Liver and Brain Metastasis	Vinorelbine+Doxorubicin+Cisplatin, Pemetrexed+Carboplatin+Bevacizumab, Icotinib+Docetaxel+Gemcitabine	B05	15/07/31	26496		
				T05	15/07/10	170.4		
				C05	15/07/31	180.8	2000	90.4
