# Supplementary material for: Cell-Free DNA Provides a Good Representation of the Tumor Genome Despite Its Biased Fragmentation Patterns
Source: PLoS One. 2017 Jan 3;12(1):e0169231. doi: 10.1371/journal.pone.0169231 (PMC5207727; doi:10.1371/journal.pone.0169231)
Supplement: S1 Fig — (A) Fragment size distribution of DNA extracted from blood samples. (B) Fragment size distribution of DNA extracted from fresh tumor, FFPE, or the cell portion of body fluid effusion samples. (C) Fragment size distribution of DNA extracted from plasma or the liquid fraction of body fluid effusion samples. This graph displays size distribution up to 400 bp. (D) Fragment size distribution of all cfDNA samples pooled together. Vertical dashed lines mark local maxima. (PDF) [file pone.0169231.s002.pdf]

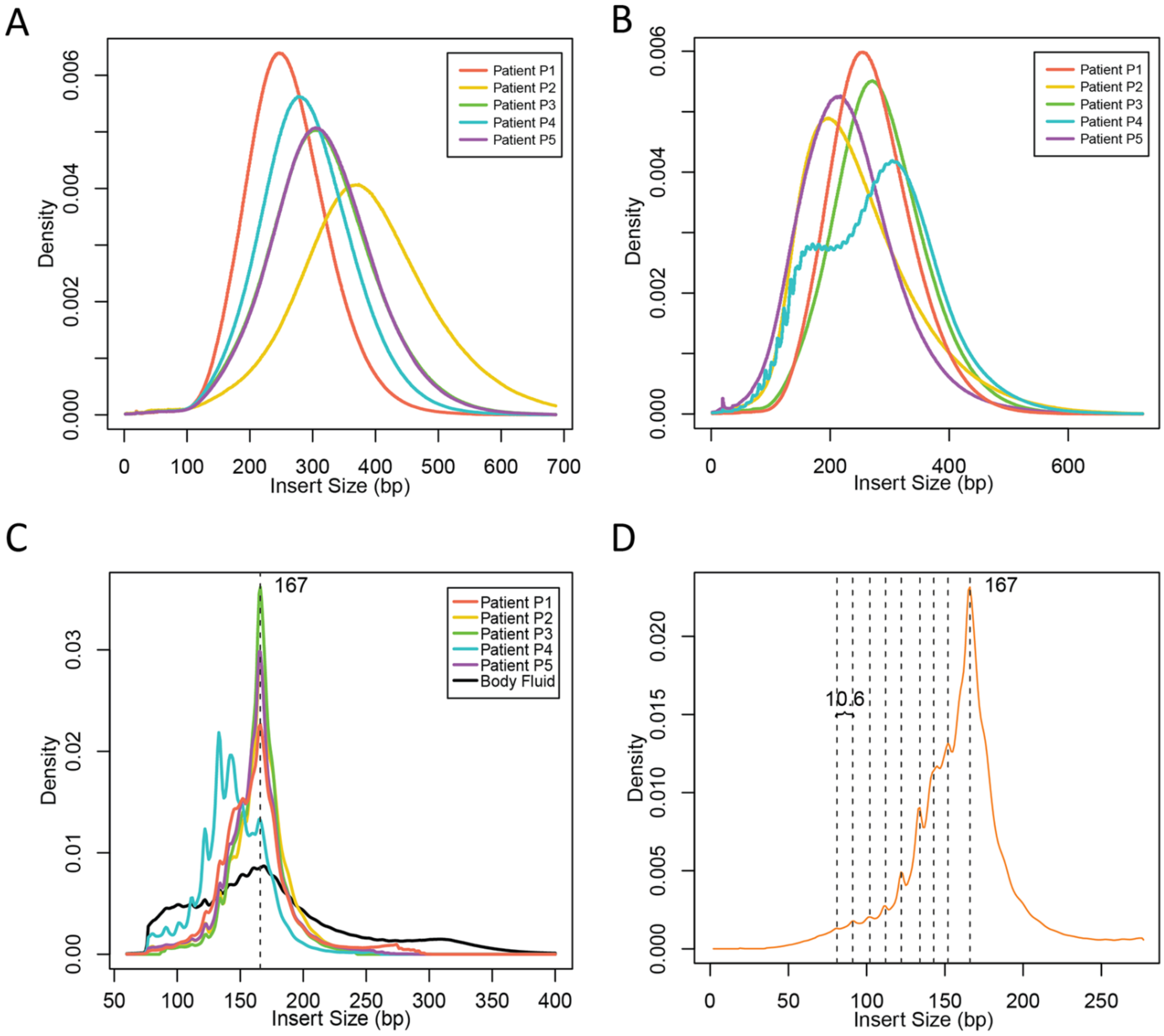

S1 Fig. WGS insert size distribution

A. Fragment size distribution of DNA extracted from blood samples.

B. Fragment size distribution of DNA extracted from fresh tumor, FFPE, or the cell portion of body fluid effusion samples.

C. Fragment size distribution of DNA extracted from plasma or the liquid fraction of body fluid effusion samples. This graph displays size distribution up to 400 bp.

D. Fragment size distribution of all cfDNA samples pooled together. Vertical dashed lines mark local maxima.
