# Supplementary material for: Cell-Free DNA Provides a Good Representation of the Tumor Genome Despite Its Biased Fragmentation Patterns
Source: PLoS One. 2017 Jan 3;12(1):e0169231. doi: 10.1371/journal.pone.0169231 (PMC5207727; doi:10.1371/journal.pone.0169231)
Supplement: S2 Fig — Plots showing the size and position of nucleotide fragments not covered in any of the (A) blood samples, (B) tumor samples, or (C) cfDNA samples. The x-axis displays genomic position. The y-axis displays the length of the no-coverage fragments in log10 scale. Vertical gray dashed lines marked the boundary between chromosomes. Sex chromosomes were excluded from this graph. (PDF) [file pone.0169231.s003.pdf]

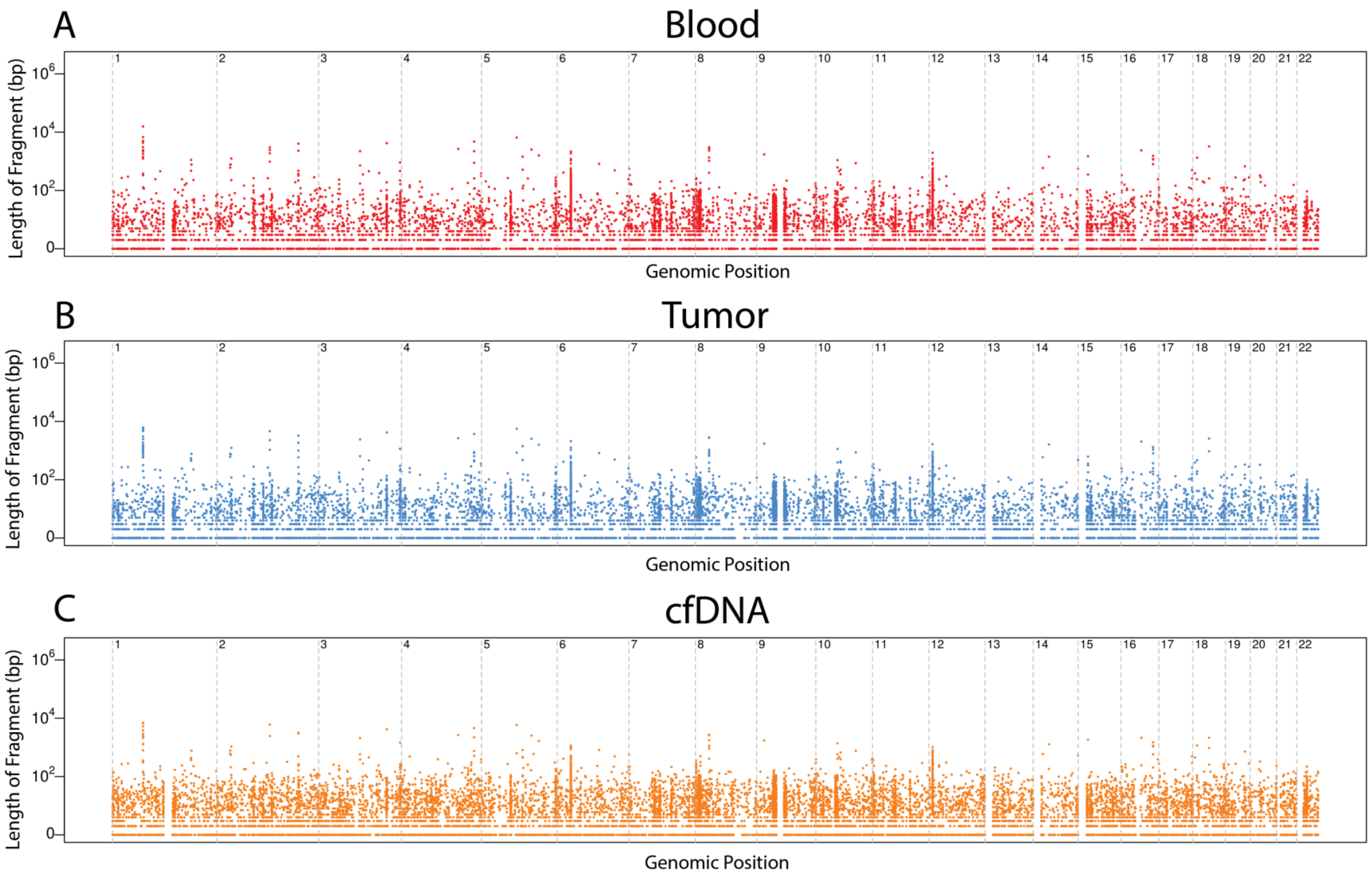

S2 Fig. Position and size of fragment not covered.

Plots showing the size and position of nucleotide fragments not covered in any of the A) blood samples, B) tumor samples, or C) cfDNA samples. The x-axis displays genomic position. The y-axis displays the length of the no-coverage fragments in $\log_{10}$ scale. Vertical gray dashed lines marked the boundary between chromosomes. Sex chromosomes were excluded from this graph.
