# Supplementary material for: Cell-Free DNA Provides a Good Representation of the Tumor Genome Despite Its Biased Fragmentation Patterns
Source: PLoS One. 2017 Jan 3;12(1):e0169231. doi: 10.1371/journal.pone.0169231 (PMC5207727; doi:10.1371/journal.pone.0169231)
Supplement: S3 Fig — GC content and read counts in each consecutive non-overlapping 10kbp window of the human reference genome hg19 were calculated. After removing regions with extreme read count values (> 99.9% percentile), LOESS was fitted to the scatterplot. Each graph demonstrates the read count spanning 25% to 70% GC content of a sample before or after the normalization. Red line represents the fitted LOESS model. (PDF) [file pone.0169231.s004.pdf]

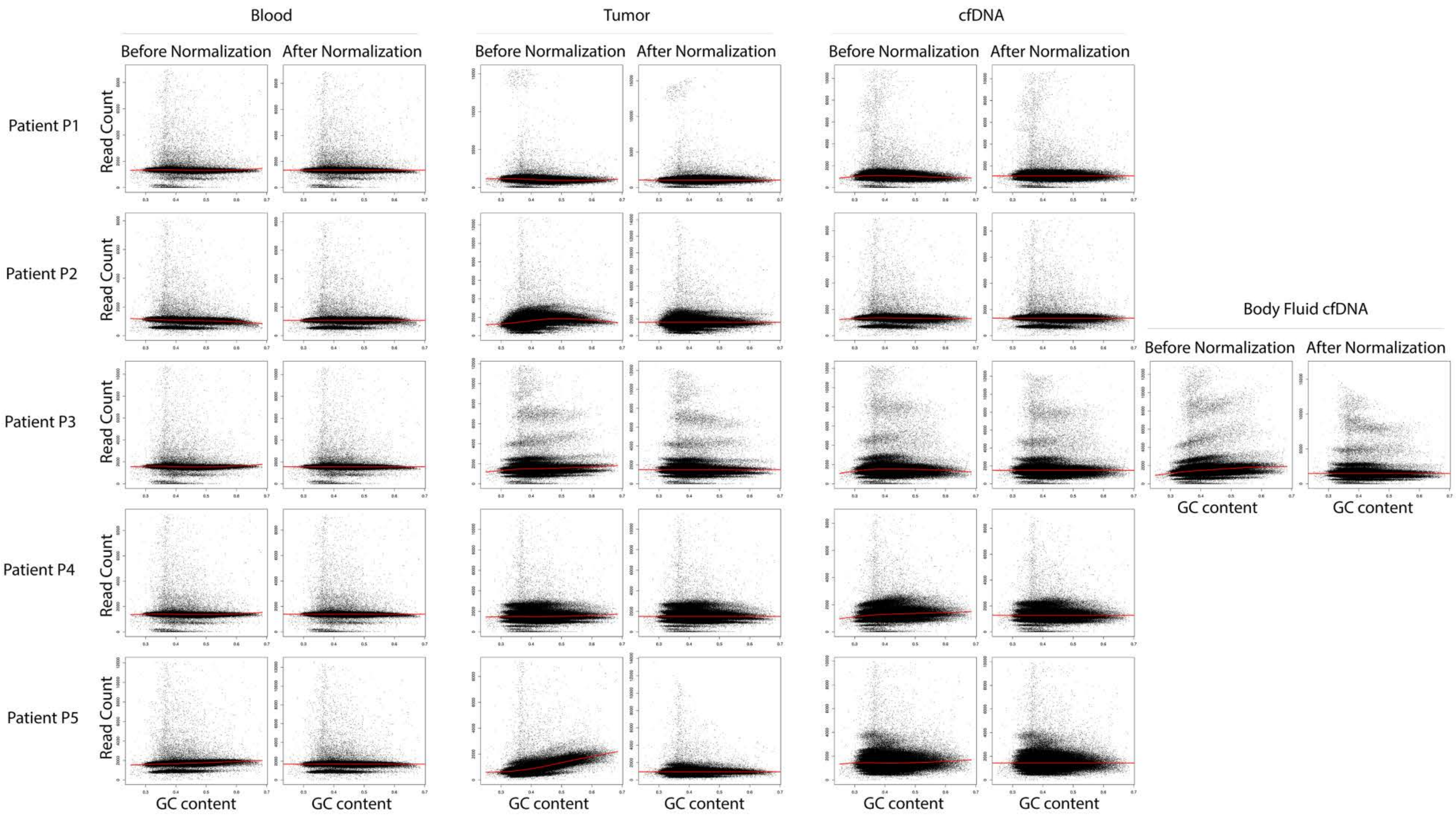

S3 Fig. Read count normalization to GC content by locally weighted scatterplot smooth (LOESS).

GC content and read counts in each consecutive non-overlapping 10k bp window of the human reference genome hg19 were calculated. After removing regions with extreme read count values (> 99.9% percentile), LOESS was fitted to the scatterplot. Each graph demonstrates the read count spanning 25% to 70% GC content of a sample before or after the normalization. Red line represents the fitted LOESS model.
