# Supplementary material for: Cell-Free DNA Provides a Good Representation of the Tumor Genome Despite Its Biased Fragmentation Patterns
Source: PLoS One. 2017 Jan 3;12(1):e0169231. doi: 10.1371/journal.pone.0169231 (PMC5207727; doi:10.1371/journal.pone.0169231)
Supplement: S4 Fig — The human reference genome hg19 was divided into consecutive non-overlapping 10kbp windows. Sequencing read count mapped to each window were tallied. After removing sex chromosomes and regions with extreme read count values (>99.9% percentile) and normalizing read count to GC content, pair-wise calculation of Spearman correlation coefficient between the CNV profiles was performed. The results were plotted in this heat map. The same color was used to label all samples collected from the same patient. (PDF) [file pone.0169231.s005.pdf]

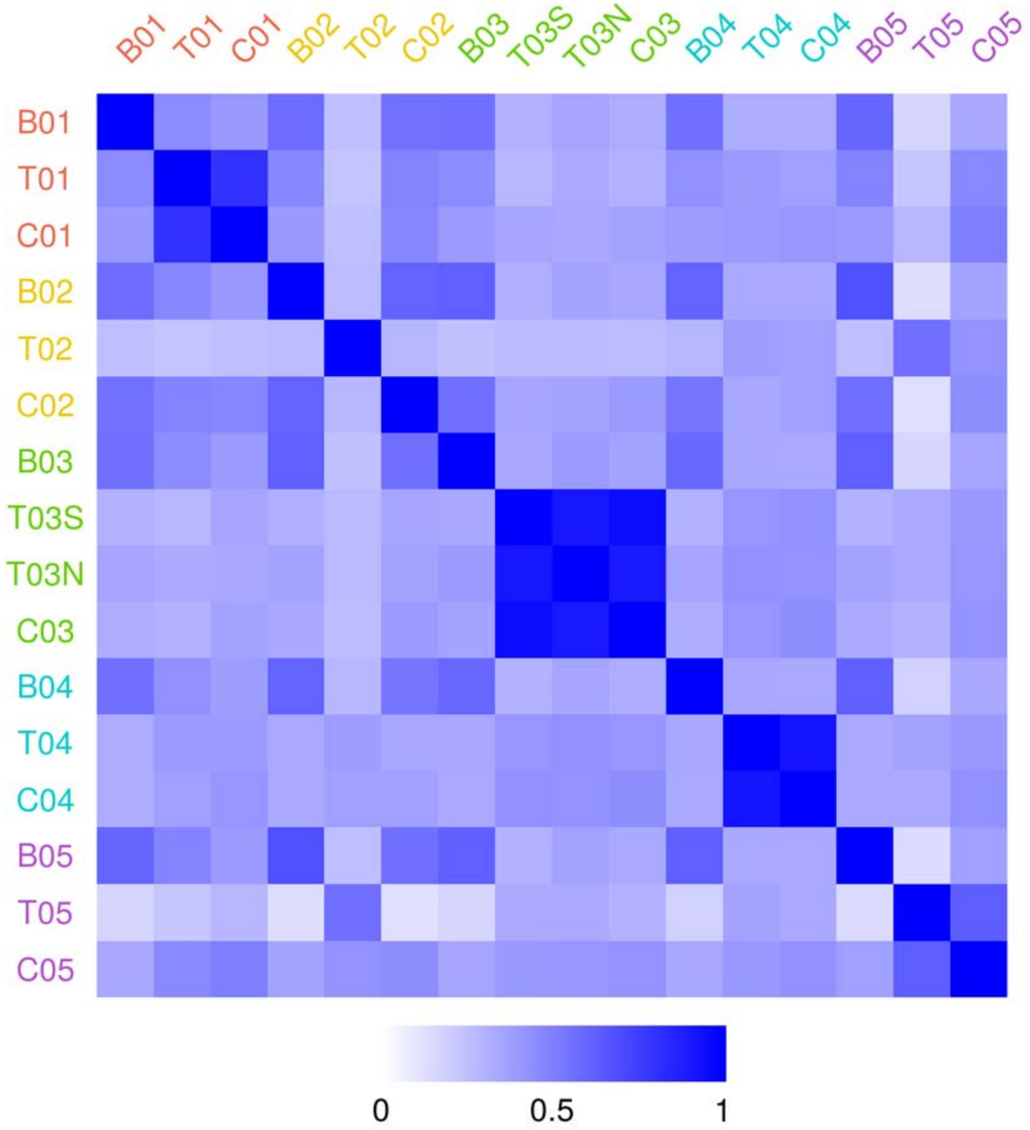

S4 Fig. Similarity between the CNV profiles.

The human reference genome hg19 was divided into consecutive non-overlapping 10k bp windows. Sequencing read count mapped to each window were tallied. After removing sex chromosomes and regions with extreme read count values (>99.9% percentile) and normalizing read count to GC content, pair-wise calculation of Spearman correlation coefficient between the CNV profiles was performed. The results were plotted in this heat map. The same color was used to label all samples collected from the same patient.
