# Supplementary material for: Cell-Free DNA Provides a Good Representation of the Tumor Genome Despite Its Biased Fragmentation Patterns
Source: PLoS One. 2017 Jan 3;12(1):e0169231. doi: 10.1371/journal.pone.0169231 (PMC5207727; doi:10.1371/journal.pone.0169231)
Supplement: S5 Fig — Coverage depth at each nucleotide normalized to regional mean depth within ±1000bp of the TSS and ±500bp of the ESS and EES in randomly selected samples from the 1000 Genome Project. Each grey line represents a sample. Red lines represent the mean value of all samples. (PDF) [file pone.0169231.s006.pdf]

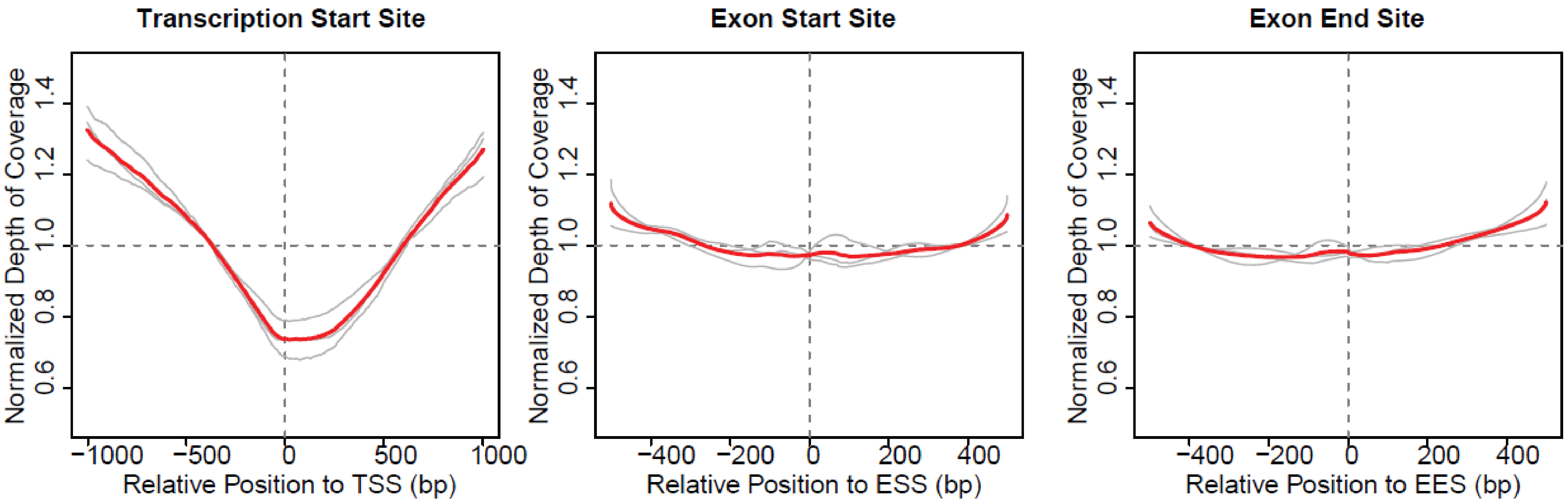

S5 Fig. Normalized coverage depth near the TSS, ESS, and EES in the 1000 Genome Project.

Coverage depth at each nucleotide normalized to regional mean depth within ±1000bp of the TSS and ±500bp of the ESS and EES in randomly selected samples from the 1000 Genome Project. Each grey line represents a sample. Red lines represent the mean value of all samples.
